# Complete Genome Sequencing of a G3P[14] Rabbit Rotavirus

**DOI:** 10.3390/ani15111548

**Published:** 2025-05-25

**Authors:** Ahmed Hassan Omar, Francesco Pellegrini, Cristiana Catella, Georgia Diakoudi, Anna Salvaggiulo, Gaia Casalino, Elena Circella, Francesco D’Amico, Michele Schiavitto, Antonio Camarda, Michele Camero, Krisztián Bányai, Jelle Matthijnssens, Max Ciarlet, Vito Martella, Gianvito Lanave

**Affiliations:** 1 Department of Veterinary Medicine, University of Bari Aldo Moro, 70010 Valenzano, Italy; ahmed.omar@uniba.it (A.H.O.); francesco.pellegrini@uniba.it (F.P.); cristiana.catella@uniba.it (C.C.); georgia.diakoudi@uniba.it (G.D.); anna.salvaggiulo@uniba.it (A.S.); gaia.casalino@uniba.it (G.C.); elena.circella@uniba.it (E.C.); francesco.damico@izsplv.it (F.D.); antonio.camarda@uniba.it (A.C.); michele.camero@uniba.it (M.C.); vito.martella@uniba.it (V.M.); gianvito.lanave@uniba.it (G.L.); 2 Istituto Zooprofilattico Sperimentale Piemonte, Liguria e Valle d’Aosta, S.S. Genova e Portualità, Borgo Pila 39, 16129 Genova, Italy; 3 Italian Rabbit Breeders Association, ANCI, Contrada Giancola Snc, Volturara Appula, 71030 Foggia, Italy; micheleschiavitto@anci-aia.it; 4 Pathogen Discovery Group, HUN-REN Veterinary Medical Research Institute, 1143 Budapest, Hungary; 5 Department of Pharmacology and Toxicology, University of Veterinary Medicine, 1078 Budapest, Hungary; 6 National Laboratory of Virology, Szentágothai Research Centre, University of Pécs, 7622 Pecs, Hungary; 7 Department of Medical Biology, Medical School, University of Pécs, 7622 Pecs, Hungary; 8 Laboratory of Viral Metagenomics, Rega Institute, Department of Microbiology, Immunology and Transplantation, University of Leuven, B-3000 Leuven, Belgium; jelle.matthijnssens@kuleuven.be; 9 Clinical Development, Icosavax, Seattle, WA 98102, USA; max.ciarlet@icosavax.com

**Keywords:** rotavirus, enteritis, genome reconstruction, G3P[14], rabbit

## Abstract

Group A rotaviruses (RVAs) are a major cause of acute dehydrating diarrhea in infants and young animals worldwide. In rabbits, RVAs are associated with enteric disease, likely in combination with other pathogens. We report the identification and characterization of a lapine RVA strain in an Italian rabbit breeding farm. Increased mortality rates associated with enteric symptoms were reported in the facility in post-weaning rabbits around 40 days of age. By quantitative RT-PCR, an RVA strain was identified in the intestinal contents of deceased rabbits. A PCR-based enrichment protocol coupled with Nanopore sequencing allowed the reconstruction of the nearly complete genome of a rabbit RVA strain, Rabbit-wt/ITA/36-9/2022/G3P[14], with a genotype constellation (G3-P[14]-I2-R2-C2-M3-A9-N2-T6-E5-H3) conserved among lapine RVAs. Each of the 11 gene segments displayed high nucleotide identity and phylogenetic clustering with lapine rotavirus strains, as well as two Belgian human G3P[14] strains, which had been shown to have a zoonotic (lapine) origin. However, the NSP2 gene of strain 36-9 clustered closer with a group of rare human G3P[9] strains, suggesting a common path during their evolution. Gathering sequence data on animal RVAs is pivotal to reconstructing the history of homologous and heterologous RVAs in various mammals, including humans.

## Simple Summary

Rotaviruses are a major cause of gastroenteritis among infants and children and also infect a variety of animals. Inter-species transmission from animals to humans and among animals with exchange of genome segments via reassortment is a powerful mechanism driving rotavirus evolution. In this study, we report the detection and characterization of a rotavirus strain in an Italian rabbit breeding farm with recurring enteric disease in young rabbits. The lapine rotavirus displayed a genotype constellation shared with other G3P[14] strains described in rabbits and humans.

## 1. Introduction

Viruses belonging to the species *Rotavirus alphagastroenteritidis,* formerly known as Group A or species A rotaviruses (RVAs), are enteric pathogens and a leading cause of severe diarrhea in human infants and in young animals worldwide [1]. In humans, gastroenteritis may lead to death, mostly in undeveloped countries. Globally, RVA infection was the leading cause of diarrheal deaths, accounting for nearly 20% of infant deaths from diarrhea in 2019 [2]. RVA caused a high death burden in African, Oceanian, and South Asian Countries in the past three decades [2]. The development of four live-attenuated oral RVA vaccines (Rotarix^®^, Rotavac^®^, Rotasiil^®^, and RotaTeq^®^) with WHO prequalification and recommendations resulted in their inclusion in national immunization programs in over 120 countries [3]. However, RVA remains the major cause of severe viral diarrhea in low-income countries [4]. RVA infection in animals is also responsible for relevant financial losses due to decreased productivity of livestock animals [5].

The RVA genome consists of 11 segments of double-stranded RNA encoding six structural proteins (VP1 to VP4, VP6, and VP7) and six nonstructural proteins (NSP1 to NSP6) [6]. The two outer capsid proteins (VP7 and VP4) induce the production of neutralizing antibodies and form the basis for the G (Glycoprotein) and P (Protease-sensitive) dual (G/P) genotyping system for RVA strains [7]. This dual genotyping system is often used for RVA classification and is updated continuously by the Rotavirus Classification Working Group [8]. According to this classification system, 42 G- and 58 P genotypes have been described globally (https://rega.kuleuven.be/cev/viralmetagenomics/virus-classification/rcwg (accessed on 27 April 2025)).

A limited number of studies have investigated the molecular characteristics of RVA strains of rabbits, with only a few lapine RVA strains isolated and characterized. Lapine RVA strains typically share the G3 genotype in combination with P[22] or P[14] [9]. Thus far, the complete genome sequence (CGS) of five rabbit RVA strains has been determined, including one G3P[22] from Korea and 4 G3P[14] strains from Italy and China [10,11,12]. Two genome constellations, G3-P[14]-I2-R2-C2-M3-A9-N2-T6-E5-H3 and G3-P[22]-I2-R3-C3-M3-A9-N2-T1-E3-H3, seem conserved, with a few exceptions [10,13].

Due to the segmented nature of the viral genome, RVA genetic diversity can be generated through reassortment events involving one or multiple gene segments [14], eventually coupled with interspecies transmission among animal species and from animals to humans [15].

Heterologous RVA infections in rabbits have also been reported. One lapine strain, RVA/Rabbit-tc/NLD/K1130027/2011/G6P[11], was shown to possess the typical bovine-like RVA genome constellation, G6-P[11]-I2-R2-C2-M2-A13-N2-T6-E2-H3 [16]. A human-derived G3P[8] strain, C-3/15, has been identified from a Mexican commercial rabbitry [17].

Cases of lapine RVAs infecting humans are scarce. In 2000, the first reported case of a lapine-like G3P[14] RVA strain (RVA/Human-wt/BEL/B4106/2000/G3P[14]) infecting a child was reported in Belgium [18]. Subsequently, the strain was demonstrated to be entirely of lapine origin but still able to cause severe gastroenteritis in a child [10]. A second report of human infection by a lapine-like RVA was reported in Belgium during the 2012–2013 rotavirus season [19], with a third zoonotic infection identified in Australia in 2012 (RVA/Human-wt/AUS/RCH272/2012/G3P[14]) in a 12-year-old child hospitalized with acute gastroenteritis [14].

Low mortality rates in rabbitries are usually considered acceptable, although efforts are made to decrease mortality in young rabbits. Due to a marked increase in mortality associated with enteritis in post-weaning rabbits in a large breeding farm, we were requested to perform laboratory investigations, and we identified RVA RNA in the tested animals. In this study, we report the characterization of the rabbit RVA strain using massive sequencing. The results revealed the presence of a G3P[14] RVA strain with peculiar genomic characteristics.

## 2. Materials and Methods

### 2.1. Collection of Samples

The study was carried out in the genetic center of the Italian Rabbit Breeders Association (ANCI), in Volturara Appula, FG, Southern Italy, authorized by the Ministry of Agricultural, Food and Forestry Policies (MIPAF) for the maintenance of rabbit breeding farms. Pure Italian White, Silver, and Spotted breeds were selected and reared in the facility. A total of 30,000 rabbits were housed in 10 sheds on the farm, with about 4000 does. A closed-cycle production system was used without introducing animals from external farms. However, does were frequently moved from one cage to another or from one shed to another due to routine husbandry procedures.

Mortality rates lower than 1% due to enteric disorders were cyclically reported in the facility in post-weaning rabbits. In January 2022, an apparent 5-fold increase in mortality was registered in one shed where two groups (A and B) of rabbits of different ages were housed. In detail, rabbits of groups A and B were 65 and 44 days old, respectively. The clinical signs observed in the affected animals were diarrhea, dehydration, and death occurring within 24–48 h. At necropsy, a total of 22 rabbits, 11 from each group, were analyzed. The gross lesions, consisting of catarrhal enteritis and, often, stasis of the stomach or cecal tract, are detailed in Table S1. Samples of the intestinal content from each rabbit were collected for laboratory investigations.

### 2.2. Sample Preparation and Nucleic Acid Extraction

The 22 samples were processed for DNA/RNA extraction. Each sample was diluted with sterile PBS, homogenized at 10% by Qiagen TissueLyser (Qiagen^TM^, Hilden, Germany), and centrifuged at 16,000× *g* for 3 min, as previously described [20]. Nucleic acid extraction was carried out using the Indispin Pathogen DNA/RNA Mini Kit (Indical^®^, Leipzig, Germany) from 200 μL of the supernatants, following the manufacturer’s instructions. Nucleic acid samples were eluted (100 μL) in laboratory-grade water and stored at −80 °C until later use.

### 2.3. Quantitative Reverse Transcription PCR (qRT-PCR) Specific for RVA

Samples from group A (*n* = 11) and B (*n* = 11) were pooled and tested for RVA by quantitative RT-PCR designed on the VP2 gene (Table 1) [21]. 

Briefly, RNA denaturation with dimethylsulphoxide (DMSO, 99.7% purity) at 97 °C for 10 min was carried out by mixing 4 μL of template and 2.8 μL of DMSO. Reverse transcription was performed using the SuperScript^TM^ IV Reverse Transcriptase kit (Invitrogen^TM^, Waltham, MA, USA) on 2 μL of nucleic acid extracts in a total reaction volume of 20 μL containing 2.5 U of random hexamers, as previously reported [22]. Cycling conditions consisted of 42 °C for 30 min, followed by a denaturation step at 99 °C for 5 min. Ten μL of sample cDNA were combined with the 15-μL reaction master mix (IQ Supermix; Bio-Rad Laboratories SRL, Segrate, Italy) comprising multiple forward and reverse primers (0.6 μmol/μL of each) and 0.2 μmol/μL of degenerate MGB TaqMan probe (Table 1). Thermal cycling was set as follows: activation of iTaq DNA polymerase at 95 °C for 10 min, 45 cycles of denaturation at 95 °C for 15 s, and annealing/extension at 60 °C for 30 s.

### 2.4. Screening for Other Pathogens

Based on the gross lesions, differential diagnosis was oriented to colibacillosis and coccidiosis. Bacteriological and parasitological investigations were carried out using standard diagnostic procedures. A flotation technique was used by mixing fecal samples with a ZnSO_4_ flotation solution (10% *w*/*v*) to detect the presence of parasite eggs and oocysts. PCR assays with primers targeting the *afr2* and *eae* fimbrial genes, encoding the intimin protein of *E. coli* with thermal cycling conditions set as follows: activation of iTaq DNA polymerase at 94 °C for 5 min, 35 cycles of denaturation at 94 °C for 15 s, annealing at 60 °C for 15 s, extension at 72 °C for 10 s, and final extension at 72 °C for 10 min [23,24] (Table 1), were also performed. Nucleic acids were also tested by a PCR assay targeting 18 s rRNA of *Eimeria* spp. with thermal cycling conditions set as follows: activation of iTaq DNA polymerase at 95 °C for 10 min, 45 cycles of denaturation at 95 °C for 15 s, and annealing/extension at 60 °C for 30 s [25].

### 2.5. Amplification of Rotavirus a Genome

The complete genome sequence of an RVA strain detected in the sample with the highest viral titer (Group B, sample 36-9/2022, *Ct* = 15.33) was amplified using a previously described protocol [26,27]. Four μL of the nucleic acid samples were combined with 2.8 μL of DMSO and subjected to RNA denaturation at 97 °C for 10 min and cooling on ice for 1 min. The RT reaction was performed in a total volume of 20 μL using the SuperScript^TM^ III First-Strand Synthesis System (Thermo Fisher Scientific, Waltham, MA, USA) kit. Briefly, 6 μL of denatured RNA was combined with 1 μL of annealing buffer and 1 μL of primer mix containing 3 pmol/µL final concentration of each primer unRAf1, unRAf2, unRAf3, unRAr1, unRAr2, and unRAr3 (Table 1) complementary to the highly conserved regions of the 5′- and 3′-ends of the RVA gene segments [27]. This reaction mixture, also mentioned as Mix 1, was incubated at 65 °C for 5 min and cooled for 1 min on ice. Mix 1 was combined with 10 μL of 2× First Strand reaction and 2 μL of SS-RNase out and incubated at 50 °C for 50 min and 85 °C for 5 min. All primers for RT were extended at the 5′-end with a 20-nt sequence for binding to the universal Up primer [26], together used as forward and reverse primers for PCR (Table 1). Amplification of all RVA gene segments was performed using the “universal” primer Up [26,27] (Table 1). The reaction was carried out using LA PCR Kit, Version 2.1 (TaKaRa Bio Europe S.A.S., Saint-Germain-en-Laye, France) in a total volume of 50 μL containing cDNA, TaKaRa LA Taq DNA polymerase (2.5 units), LA PCR Buffer II with Mg2+ (final concentration at 3.5 mM), dNTPs (final concentration of 1 mM each), and universal primer Up at 1 uM. Initial denaturation was performed at 95 °C for 2 min, followed by 1 cycle of denaturation at 94 °C for 30 s, annealing at 42 °C for 30 s, and extension at 68 °C for 20 min. Subsequently, a further 10 cycles of denaturation at 94 °C for 30 s, annealing at 55 °C for 30 s, and extension at 68 °C for 10 min were carried out. The last step of the thermal file comprised an additional 30 cycles of denaturation at 94 °C for 30 s, annealing at 65 °C for 30 s and annealing at 68 °C for 30 s, and a final extension at 68 °C for 10 min.

**Table 1:** Oligonucleotides used in this study.

Pathogen	Target Gene	Assay	Primer/Probes	Sequence 5′-3′	Reference
RVA	VP2	qRT-PCR	Vp2f1	TCTGCAGACAGTTGAACCTATTAA	[21]
			Vp2f2	CAGACACGGTTGAACCCATTAA	
			Vp2f3	TCGGCTTGATACAGTAGAACCTATAAATG	
			Vp2f4	TGTCAGCTGATACAGTAGAACCTATAAATG	
			Vp2f5	TCAGCTGAC ACAGTAGAACCTATAAATG	
			Vp2R1	GTTGGCGTTTACAGTTCGTTCAT	
			Vp2R2	GTTGGCGTCTACAATTCGTTCAT	
			VP2-probe	FAM-ATGCGCATRTTRTCAAAHGCAA-MGB-NFQ	
RVA	Complete genome	RT	unRAf1	GCCGGAGCTCTGCAGAATTCGGCTWTWAAA	[27]
			unRAf2	GCCGGAGCTCTGCAGAATTCGGCTTTTTTT	
			unRAf3	GCCGGAGCTCTGCAGAATTCGGCTTTTAAT	
			unRAr1	GCCGGAGCTCTGCAGAATTCGGTCAYATC	
			unRAr2	GCCGGAGCTCTGCAGAATTCGGTCACAWA	
			unRAr3	GCCGGAGCTCTGCAGAATTCAGCCACATG	
	Universal	PCR	Up	GCCGGAGCTCTGCAGAATTC	[26]
*E. coli*	*eae*	PCR	ECW1 s	TGCGGCACAACAGGCGGCGA	[23]
			ECW2 as	CGGTCGCCGCACCAGGATTC	
	*afr2*	PCR	AF/R2-F	AAGTTAGGGGACGCCATTAC	[24]
			AF/R2-R	CCAGGACTTATTCTGACCAG	
*Eimeria* spp.	18srRNA	PCR	1FE	TACCCAATGAAAACAGTTT	[25]
			4RB	CGTCTTCAAACCCCCTACTG	

### 2.6. Oxford Nanopore Technologies (ONT) Sequencing

Length distributions of the obtained fragments of the amplicons were evaluated by an A2100 Bioanalyzer (Agilent Technologies, Santa Clara, CA, USA) with a High Sensitivity DNA chip, while DNA concentration was evaluated in a Qubit 4.0 Fluorometer using a Qubit dsDNA HS kit (Invitrogen, Life Technologies, Milan, Italy), both according to the manufacturer’s instructions. The Ligation Sequencing Kit V14 (SQK-LSK114) (Oxford Nanopore Technologies, ONT^TM^, Oxford, UK) was used to prepare libraries, which were purified using Agencourt AMPure XP magnetic beads (Beckman Coulter^TM^, Indianapolis, IN, USA). The libraries were pooled and sequenced for 24 h using the MinION Flongle Flow Cell (R10.4.1) FLO-FLG001 on the MinION-Mk1C device (ONT^TM^, Oxford, UK) provided with the Flongle adapter.

### 2.7. Sequence and Phylogenetic Analyses

Sequencing reads produced by the Nanopore platform were analyzed using the online bioinformatics server Genome Detective Virus Tool, v 2.48 [28]. The bioinformatic pipeline for quality control, adaptor trimming, and de novo assembly of reads using SPAdes used here has been previously reported [29]. In parallel, FastQ MinION files were also subjected to quality control, trimming, and reference assembly by Minimap2 implemented in Geneious Prime software, v2024.0. Open reading frame predictions and annotations were performed in Geneious Prime software, v 2024.0. The online tool Blast Nucleotide (BLASTn, https://blast.ncbi.nlm.nih.gov, accessed on 27 April 2025) was employed using the default values to find homologous hits based on nucleotide (nt) identity. The whole genome sequence obtained was deposited in the GenBank database from the National Center for Biotechnology Information (NCBI) database.

The obtained sequences were aligned with cognate rotavirus sequences recovered from the NCBI database using the MAFFT plugin implemented in Geneious Prime software, v 2024.0. The most reliable substitution model for the phylogeny was estimated by using “Find the best protein DNA/Protein Models” comprised in MEGA X, v 10.0.5 software [30]. The maximum-likelihood method, the Tamura 3-parameter model, a discrete gamma distribution, and invariant sites to model evolutionary rate differences among sites (6 categories) were selected. One thousand replicates were used for statistical support.

## 3. Results

RVA was detected at a cycle threshold (Ct) equal to 42.66 in pool A and at a Ct equal to 25.88 in pool B. Samples of pools A and B were also tested individually, yielding 6/11 RVA-positive animals in group A (with *Ct* values ranging from 35.24 to 38.56) and 9/11 (81.8%) RVA-positive animals in group B (with *Ct* values ranging between 15.33 and 31.34). Microbiological and parasitological investigations also revealed the presence of biotype 31 of *E. coli* with virulence-associated genes (*eae* and *afr2*) and the presence of *Eimeria* spp. (Table S1).

The RVA-positive sample with the lowest Ct (sample 36-9/2022, *Ct* value = 15.33) was subjected to an amplification protocol to recover the complete genome of the RVA strain [26,27]. Nanopore sequencing allowed the reconstruction of 11 genome segments of strain RVA/Rabbit-wt/ITA/36-9/2022/G3P[14]. The nearly complete genome was assembled using a total of 1224,360 reads, with a 67,318 mean depth coverage (range 8050–244,403) (Table 2). The results obtained using the research bioinformatic pipeline of the online software Genome Detective Virus Tool and the Minimap2 tool version 2.17 implemented in the Geneious Prime software were comparable.

The novel RVA strain Rabbit-wt/ITA/36-9/2022/G3P[14] displayed the same genotype constellation G3-P[14]-I2-R2-C2-M3-A9-N2-T6-E5-H3 as that described in human RVA strains BE5028 and B4106 from Belgium and in a rabbit RVA strain 30-96 from Italy [18,19,31] (Table 3).

Gene segments 1, 2, and 10 encoding VP1, VP2, and NSP4 genes, respectively, of the RVA strain Rabbit-wt/ITA/36-9/2022/G3P[14] detected in this study displayed the highest nt identity in the NCBI database to the Belgian RVA strain Human-wt/BEL/B4106/2000/G3P[14]. Gene segments 3 to 7 and 9 and 11, encoding VP3, VP4, NSP1, VP6, NSP3, VP7, and NSP5 genes, respectively, were closely related to the Belgian RVA strain Human-wt/BEL/BE5028/2012/G3P[14]. Gene segment 8, coding the NSP2, was more related to the RVA strain Human-wt/RUS/Omsk08-442/2008/G3P[9] isolated in Russia (Table 4).

**Figure 1:**
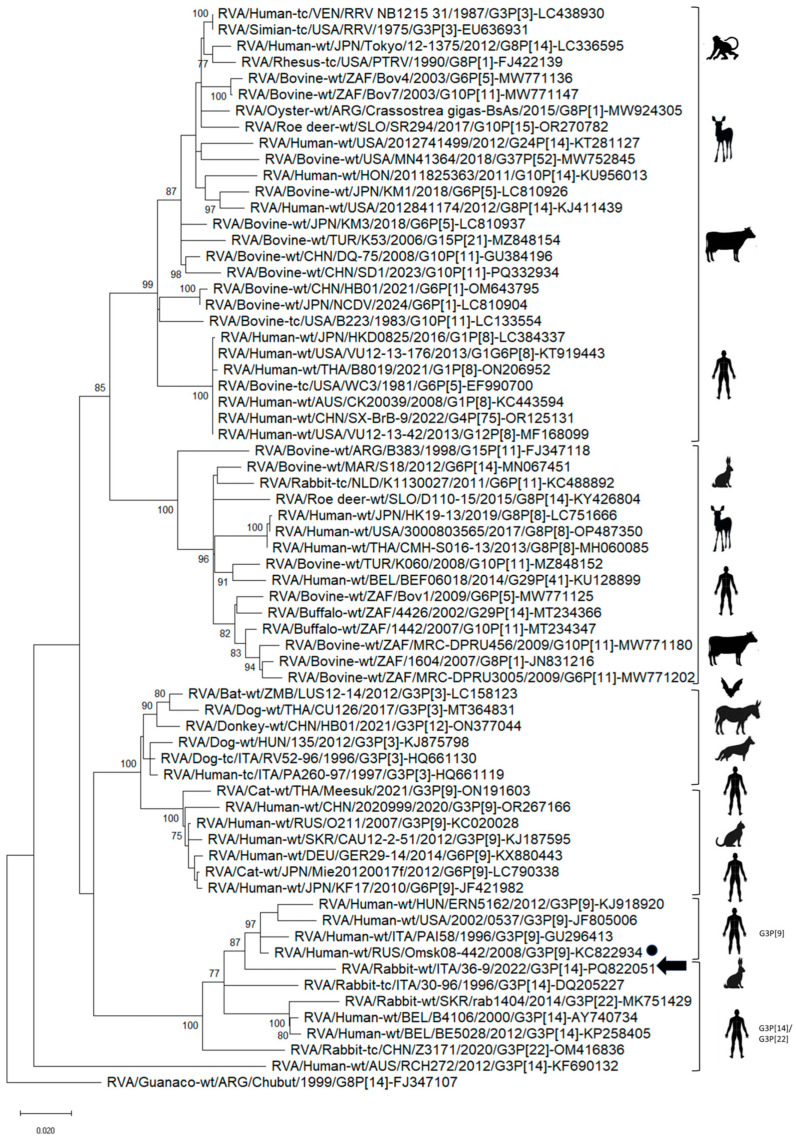
NSP2 gene-based phylogenetic tree. The rotavirus A (RVA) strain identified in this study was compared with 66 cognate sequences of RVA strains of genotype N2 retrieved from the NCBI database. The black arrow indicates the lapine RVA strain detected in this study. Black circles indicate the reference strain with the highest nt identity to Rabbit-wt/ITA/36-9/2022/G3P[14] according to Table 4. The scale bar represents the number of nt substitutions per site.

In the phylogenetic analysis based on the segments 1–7 and 9–11, strain Rabbit-wt/ITA/36-9/2022/G3P[14] was intermingled with lapine RVAs and with lapine-like human RVA strains (Figure S1). A phylogenetic tree was generated using a subset of RVA NSP2 gene sequences, selected based on the results of database interrogation with BLAST Nucleotide (interrogation on 27 April 2025) and including only NSP2 sequences of N2 genotype (Figure 1). In this analysis, the RVA strain Rabbit-wt/ITA/36-9/2022/G3P[14] clustered with a group encompassing human and rabbit G3P[14] and G3P[22] RVAs (93.4 to 94.3% nt identity) and a group formed by G3P[9] RVAs detected in the USA, Europe, and Russia (94.7 to 96.2% nt identity).

The nt sequences of the 11 genome segments of the RVA strain Rabbit-wt/ITA/36-9/2022/G3P[14] were deposited in GenBank under accession numbers PQ822044 to PQ822054.

## 4. Discussion

RVA infection is endemic in rabbitries, and antibodies to RVA are common in rabbits after 4 months of age, up to 98% of subjects [32,33,34,35,36,37]. Since passively transferred antibodies protect young rabbits up to 2 months of age, RVA-associated disease is usually described after weaning in 1- to 3-month-old animals [33,36,37]. In experimental infection of rabbits with RVA, the enteric disease seems age-restricted to the neonatal period (≤2 week) [33], although strain-related variations in terms of virulence or tropism could result in different patterns of infection/disease [38]. Epidemiological investigations in rabbitries in Mexico, the USA, and Italy hint at a possible association with enteric signs, with the RVA prevalence ranging between 17.6 and 25% in young rabbits with enteritis [9,39,40]. In a comprehensive virological survey of European wild rabbits (*Oryctolagus cuniculus algirus*) in the Iberian Peninsula [41], RVA RNA was detected in 48.1% (13 out of 27 tested) of carcasses found dead in the field (both adult and juvenile rabbits), but it was not detected in 32 overtly healthy hunted wild rabbits.

Overall, RVA could play a role in the etiology of enteric disease by either exerting direct pathogenic activity or in synergism with other pathogens (i.e., *Clostridium difficile*, *Clostridium spiroforme*, or *E. coli*) [33]. In this study, RVA was identified in rabbits deceased with enteric disease. In the rabbit flocks, an increase in mortality rates associated with enteric disorders above the expected threshold in post-weaning rabbits was observed. Laboratory investigations identified *E. coli* strains with virulence-associated genes (*eae* and *afr2*) [42] and parasites (*Eimeria* spp.) [43]. Therefore, the exact pathogenic role of RVA in this enteric form remains unclear. In addition, stasis of the stomach and cecal tract, which is often due to a low rate of fiber in the feed, was also observed, noting a multi-factorial etiology in the syndrome. Managing enteric diseases in rabbits is complicated, and developing effective control and prevention strategies in breeding facilities against enteric pathogens is crucial.

Genotyping of VP4 and VP7 antigens has revealed that rabbit RVA strains have a G3 VP7 type in combination with either [P14] or P[22] VP4 antigens [18,44]. In a large-scale epidemiological study on 350 stool samples from 25 rabbitries from 1998 to 2004 in Italy, the vast majority of the strains were typed as G3P[22], while only one RVA strain was G3P[14], and two samples contained a mixed G3 P[14] + [22] RVA infection [9]. This suggests that G3P[14] strains are not common or that there may be geographical/temporal variations. Rabbit RVA strains with either G3P[14] or G3P[22] genotypes have been identified in several countries, including South Korea, China, Canada, Italy, and Japan [10,11,12,13,19,45].

Since information on the CGS of rabbit RVAs is still limited, we generated the CGS of a rabbit RVA identified in this study. Ten out of eleven segments of strain Rabbit-wt/ITA/36-9/2022/G3P[14] were highly similar to lapine-like RVA strains identified in Belgium in human patients [10,19] and to other lapine RVAs. However, the NSP2 genome segment displayed the highest nt identity to the RVA strain Human-wt/RUS/Omsk08-442/2008/G3P[9] identified from a human patient in Russia in 2008 [46] (Table 4). A phylogenetic tree was generated using a subset of RVA NSP2 genes, selected based on the results of database interrogation with BLAST. In this analysis, strain Rabbit-wt/ITA/36-9/2022/G3P[14] appeared intermingled between rabbit RVAs and a group of human G3P[9] RVA strains, suggesting that the NSP2 gene of these human G3P[9] viruses likely shared an evolutionary pathway with rabbit RVAs, implying genetic drift and reassortments (Figure 1). Interestingly, for some of these G3P[9] human RVAs, CGS data have been generated, revealing an unusual pattern of reassortment in the genome, shared with RVAs of ruminants and cats [47,48,49] and markedly different from AU-1-like G3P[9] human RVAs (Table 3).

Heterologous RVAs can infect rabbits under experimental conditions, although the infection is usually not transmitted horizontally [32,33]. In 2013, the isolation of a bovine-like RVA strain (RVA/Rabbit-tc/NLD/K1130027/2011/G6P[11]) was reported in the Netherlands in rabbits of a laboratory animal facility. Upon CGS analysis, the virus was shown to be related in all genes to either RVAs of ruminants or to bovine-like G6 human RVAs [16]. Also, a human-like G3P[8] strain was identified from a rabbit production farm, with enteric disease in 40- to 60-day-old animals [17]. These findings reinforce the notion that the evolution of human RVAs is tightly intermingled with that of animal RVAs, with a 2-way flow exchange of genetic material, implying forward and backward transmission events among different animal species. Of note, it is known that rabbits can host other viruses with zoonotic potential, as approximately 0.5% (5/919) of human cases of hepatitis E in France in 2015–2016 were caused by rabbit hepatitis E viruses [50].

## 5. Conclusions

In conclusion, we report the identification of a G3P[14] rabbit RVA strain identified in a large breeding farm with recurring problems of enteric disease in young rabbits.

Our study revealed that ten out of eleven segments of strain Rabbit-wt/ITA/36-9/2022/G3P[14] were highly similar to lapine-like RVA strains identified in human patients and to other lapine RVAs [43,45]. Also, the NSP2 gene was similar to a group of unusual human G3P[9] viruses. This would suggest that the NSP2 gene of these human G3P[9] viruses likely shared an evolutionary pathway with rabbit RVAs. Surveillance and studies of animal viruses are relevant to improving animal health and understanding their diversity and evolution but are also relevant to pursuing the principles of the One Health paradigm to decipher the origin of viruses with zoonotic potential.

## Figures and Tables

**Table 2:** Number of reads obtained and depth and genome coverage per genome segment for strain RVA/Rabbit-wt/ITA/36-9/2022/G3[P14].

Gene	Segment	Length (nt *)	Reads (Nr **)	Coverage Depth	NCBI Accession
VP1	1	3302/3302	61,226	37,211	PQ822044
VP2	2	2687/2690	113,982	85,274	PQ822045
VP3	3	2591/2591	9989	8050	PQ822046
VP4	4	2361/2362	14,713	13,158	PQ822047
NSP1	5	1597/1611	25,116	35,791	PQ822048
VP6	6	1356/1356	37,152	55,260	PQ822049
NSP3	7	1072/1104	129,942	244,403	PQ822050
NSP2	8	1059/1059	132,699	25,339	PQ822051
VP7	9	1062/1062	64,508	129,080	PQ822052
NSP4	10	751/751	13,765	37,195	PQ822053
NSP5	11	1035/1035	621,268	69,740	PQ822054

RNA-dependent RNA polymerase (VP1); capsid protein (VP2); capping enzyme (VP3); spike protein (VP4); nonstructural RNA-binding protein (NSP1); major inner capsid protein (VP6); translational regulator (NSP3); multifunctional RNA chaperone (NSP2); outer capsid protein (VP7); viral enterotoxin (NSP4); viral phosphoprotein (NSP5). * nt = nucleotide. ** Nr = number of reads

**Table 3:** Comparison of the genotype constellation of the rotavirus A strain detected in this study with other rotavirus A strains, based on BLASTn database interrogation (accessed on 27^th^ April 2025). Light gray shade is used to indicate the same genotype. Dark grey shade indicates the highest nt identity.

RVA Strain	VP7	VP4	VP6	VP1	VP2	VP3	NSP1	NSP2	NSP3	NSP4	NSP5/6
Human-tc/USA/Wa/1974/G1P[8]	G1	P[8]	I1	R1	C1	M1	A1	N1	T1	E1	H1
Human-tc/USA/DS-1/1976/G2P[4]	G2	P[4]	I2	R2	C2	M2	A2	N2	T2	E2	H2
Human-tc/JPN/AU-1/1982/G3P[9]	G3	P[9]	I3	R3	C3	M3	A3	N3	T3	E3	H3
Human-wt/BEL/BE5028/2012/G3P[14]	G3	P[14]	I2	R2	C2	M3	A9	N2	T6	E5	H3
Human-wt/BEL/B4106/2000/G3P[14]	G3	P[14]	I2	R2	C2	M3	A9	N2	T6	E5	H3
Human-wt/AUS/RCH272/2012/G3P[14]	G3	P[14]	I2	R3	C3	M3	A9	N2	T6	E2	H3
Human-wt/RUS/Omsk08-442/2008/G3P[9]	G3	P[9]	I2	ND	ND	ND	ND	N2	ND	ND	ND
Human-wt/HUN/ERN5162/2012/G3P[9]	G3	P[9]	I2	R2	C2	M2	A3	N2	T3	E3	H3
Human-wt/ITA/PA158/1996/G3P[9]	G3	P[9]	I2	R2	C2	M2	A3	N2	T6	E2	H3
Human-wt/USA/0537/2002/G3P[9]	G3	P[9]	I2	R2	C2	M2	A3	N2	T1	E2	H3
**Rabbit-wt/ITA/36-9/2022/G3P[14]**	**G3**	**P[14]**	**I2**	**R2**	**C2**	**M3**	**A9**	**N2**	**T6**	**E5**	**H3**
Rabbit-tc/ITA/30-96/1996/G3P[14]	G3	P[14]	I2	R2	C2	M3	A9	N2	T6	E5	H3
Rabbit-tc/CHN/N5/1992/G3P[14]	G3	P[14]	I17	R3	C3	M3	A9	N1	T1	E3	H2
Rabbit-tc/NLD/K1130027/2011/G6P[11]	G6	P[11]	I2	R2	C2	M2	A13	N2	T6	E2	H3
Rabbit-tc/CHN/Z3171/2020/G3P[22]	G3	P[22]	I2	R3	C3	M3	A9	N2	T1	E3	H3
Rabbit-wt/SKR/Rab1404/2014/G3P[22]	G3	P[22]	I2	R3	C3	M3	A9	N2	T3	E3	H3
Rabbit-wt/MEX/C-3-15/2015/G3P[8]	G3	P[8]	ND	ND	ND	ND	ND	ND	ND	ND	ND
Cat-wt/ITA/BA222/2005/G3P[9]	G3	P[9]	I2	R2	C2	M2	A3	N1	T3	E2	H3
Cat-tc/AUS/Cat97/1984/G3P[3]	G3	P[3]	I3	R3	C2	M3	A9	N2	T3	E3	H6
Cat-tc/JAP/FRV-1/1982/G3P[9]	G3	P[9]	I3	R3	C3	M3	A3	N3	T3	E3	H3
Dog-tc/ITA/RV198-95/1995/G3P[3]	G3	P[3]	I3	R3	C2	M3	A9	N2	T3	E3	H6
Cow-tc/USA/NCDV/1971/G6P[1]	G6	P[1]	I2	R2	C2	M2	A3	N2	T6	E2	H3
Cow-wt/TUR/K53/2006/G15P[21]	G15	P[21]	I2	R2	C2	M2	A13	N2	T9	E2	H3
Pig-tc/USA/Gottfried/1975/G4P[6]	G4	P[6]	I1	R1	C1	M1	A8	N1	T1	E1	H1
Pig-tc/USA/OSU/1975/G5P[7]	G5	P[7]	I5	R1	C1	M1	A1	N1	T1	E1	H1

In bold genotype constellation of the rotavirus A strain detected in this study is shown.

**Table 4:** RVA strains with the highest nt identity per segment to Rabbit-wt/ITA/36-9/2022/G3P[14], determined by BLASTn (consulted on 24 January 2025).

Gene	Segment	Genotype	Reference Strain	Accession No	% nt Identity
VP1	1	R2	RVA/Human wt/BEL/B4106/2000/G3P[14]	AY740741	93.7%
VP2	2	C2	RVA/Human-wt/BEL/B4106/2000/G3P[14]	AY740740	97.4%
VP3	3	M3	RVA/Human-wt/BEL/BE5028/2012/G3P[14]	KP258400	98.9%
VP4	4	P[14]	RVA/Human-wt/BEL/BE5028/2012/G3P[14]	KP258401	98.1%
NSP1	5	A9	RVA/Human-wt/BEL/BE5028/2012/G3P[14]	KP258404	98.3%
VP6	6	I2	RVA/Human-wt/BEL/BE5028/2012/G3P[14]	KP258402	97.9%
NSP3	7	T6	RVA/Human-wt/BEL/BE5028/2012/G3P[14]	KP258406	98.3%
NSP2	8	N2	RVA/Human-wt/RUS/Omsk08-442/2008/G3P[9]	KC822934	96.2%
VP7	9	G3	RVA/Human-wt/BEL/BE5028/2012/G3P[14]	KP258403	96.7%
NSP4	10	E5	RVA/Human-wt/BEL/BE5028/2012/G3P[14]	KP258407	98.1%
NSP5	11	H3	RVA/Human-wt/BEL/BE5028/2012/G3P[14]	KP258408	98.5%

## Data Availability

The data that support the findings are contained in the paper.

## Supplementary Materials

The following supporting information can be downloaded at: https://www.mdpi.com/article/10.3390/ani15111548/s1. Table S1: Summary of the gross pathological findings and diagnostic screening for Rotavirus A (RVA), *Escherichia coli* (*E. coli*) and *Eimeria* spp. for the two cohorts of post-weaning rabbits (Group A and Group B). The severity of infection induced by *Eimeria* spp. is denoted by the number of plus signs (+). Figure S1: Phylogenetic trees of RNA-dependent RNA polymerase (VP1); capsid protein (VP2); capping enzyme (VP3); spike protein (VP4); nonstructural RNA-binding protein (NSP1); major inner capsid protein (VP6); translational regulator (NSP3); multifunctional RNA chaperone (NSP2); outer capsid protein (VP7); viral enterotoxin (NSP4); viral phosphoprotein (NSP5) nucleotide sequences of the rotavirus A strain Rabbit-wt/ITA/36-9/2022/G3P[14] compared with cognate sequences retrieved from GenBank database. Phylogeny of VP1 gene (**A**). Phylogeny of VP2 gene (**B**). Phylogeny of VP3 gene (**C**). Phylogeny of VP4 gene (**D**). Phylogeny of NSP1 gene (**E**). Phylogeny of VP6 gene (**F**). Phylogeny of NSP3 gene (**G**). Phylogeny of VP7 gene (**H**). Phylogeny of NSP4 gene (**I**). Phylogeny of NSP5 gene (**J**). The black arrow indicates the lapine RVA strain detected in this study. Black circles indicate the reference strain with the highest nt identity to Rabbit-wt/ITA/36-9/2022/G3P[14] according to Table 4. The scale bar represents the number of nt substitutions per site.

## References

[1] Bishop R. (2009). Discovery of rotavirus: Implications for child health. J. Gastroenterol. Hepatol..

[2] Du Y., Chen C., Zhang X., Yan D., Jiang D., Liu X., Yang M., Ding C., Lan L., Hecht R. (2022). Global burden and trends of rotavirus infection-associated deaths from 1990 to 2019: An observational trend study. Virol. J..

[3] Chen J., Grow S., Iturriza-Gómara M., Hausdorff W.P., Fix A., Kirkwood C.D. (2022). The Challenges and Opportunities of Next-Generation Rotavirus Vaccines: Summary of an Expert Meeting with Vaccine Developers. Viruses.

[4] Buttery J.P., Kirkwood C. (2021). Rotavirus vaccine implementation: Evidence to fill the gap?. Lancet Glob. Health.

[5] Tate J.E., Burton A.H., Boschi-Pinto C., Steele A.D., Duque J., Parashar U.D., WHO-coordinated Global Rotavirus Surveillance Network (2012). 2008 estimate of worldwide rotavirus-associated mortality in children younger than 5 years before the introduction of universal rotavirus vaccination programmes: A systematic review and meta-analysis. Lancet Infect. Dis..

[6] Pesavento J.B., Crawford S.E., Estes M.K., Prasad B.V. (2006). Rotavirus proteins: Structure and assembly. Curr. Top. Microbiol. Immunol..

[7] Kim H.H., Matthijnssens J., Kim H.J., Kwon H.J., Park J.G., Son K.Y., Ryu E.H., Kim D.S., Lee W.S., Kang M.I. (2012). Full-length genomic analysis of porcine G9P[23] and G9P[7] rotavirus strains isolated from pigs with diarrhea in South Korea. Infect. Genet. Evol..

[8] Matthijnssens J., Ciarlet M., McDonald S.M., Attoui H., Bányai K., Brister J.R., Buesa J., Esona M.D., Estes M.K., Gentsch J.R. (2011). Uniformity of rotavirus strain nomenclature proposed by the Rotavirus Classification Working Group (RCWG). Arch. Virol..

[9] Martella V., Ciarlet M., Lavazza A., Camarda A., Lorusso E., Terio V., Ricci D., Cariola F., Gentile M., Cavalli A. (2005). Lapine rotaviruses of the genotype P[22] are widespread in Italian rabbitries. Vet. Microbiol..

[10] Matthijnssens J., Rahman M., Martella V., Xuelei Y., De Vos S., De Leener K., Ciarlet M., Buonavoglia C., Van Ranst M. (2006). Full genomic analysis of human rotavirus strain B4106 and lapine rotavirus strain 30/96 provides evidence for interspecies transmission. J. Virol..

[11] Guo D., Liu J., Lu Y., Sun Y., Yuan D., Jiang Q., Lin H., Li C., Si C., Qu L. (2012). Full genomic analysis of rabbit rotavirus G3P[14] strain N5 in China: Identification of a novel VP6 genotype. Infect. Genet. Evol..

[12] Oem J.K., Lee S.Y., Kim Y.S., Na E.J., Choi K.S. (2019). Genetic characteristics and analysis of a novel rotavirus G3P[22] identified in diarrheic feces of Korean rabbit. Infect. Genet. Evol..

[13] Zhao Q., Liu L., Huang T., Tian Y., Guo X., Liu C., Huang B., Chen Q. (2023). Complete genomic analysis of rabbit rotavirus G3P[22] in China. Arch. Virol..

[14] Donato C.M., Manuelpillai N.M., Cowley D., Roczo-Farkas S., Buttery J.P., Crawford N.W., Kirkwood C.D. (2014). Genetic characterization of a novel G3P[14] rotavirus strain causing gastroenteritis in 12 year old Australian child. Infect. Genet. Evol..

[15] Watanabe M., Nakagomi T., Koshimura Y., Nakagomi O. (2001). Direct evidence for genome segment reassortment between concurrently-circulating human rotavirus strains. Arch. Virol..

[16] Schoondermark-van de Ven E., Van Ranst M., de Bruin W., van den Hurk P., Zeller M., Matthijnssens J., Heylen E. (2013). Rabbit colony infected with a bovine-like G6P[11] rotavirus strain. Vet. Microbiol..

[17] Reynoso-Utrera E., Bautista-Gómez L.G., Fonseca-Coronado S., Pérez-de la Rosa J.D., Rodríguez-Villavicencio V.J., Romero-Núñez C., Flores-Ortega A., Hernández-García P.A., Martínez-Castañeda J.S. (2024). New Genotype G3 P[8] of Rotavirus Identified in a Mexican Gastroenteric Rabbit. Viruses.

[18] De Leener K., Rahman M., Matthijnssens J., Van Hoovels L., Goegebuer T., van der Donck I., Van Ranst M. (2004). Human infection with a P[14], G3 lapine rotavirus. Virology.

[19] Bonica M.B., Zeller M., Van Ranst M., Matthijnssens J., Heylen E. (2015). Complete genome analysis of a rabbit rotavirus causing gastroenteritis in a human infant. Viruses.

[20] Pellegrini F., Lanave G., Caringella F., Diakoudi G., Salvaggiulo A., Cavalli A., Papaleo A., Di Martino B., Camero M., Bányai K. (2024). Identification of Recombinant Aichivirus D in Cattle, Italy. Animals.

[21] Gutiérrez-Aguirre I., Steyer A., Boben J., Gruden K., Poljsak-Prijatelj M., Ravnikar M. (2008). Sensitive detection of multiple rotavirus genotypes with a single reverse transcription-real-time quantitative PCR assay. J. Clin. Microbiol..

[22] Ndiana L.A., Lanave G., Desario C., Odigie A.E., Madubuike K.G., Lucente M.S., Ezeifeka C.A., Patruno G., Lorusso E., Elia G. (2023). Detection of Selected Canine Viruses in Nigerian Free-Ranging Dogs Traded for Meat Consumption. Animals.

[23] Wieler L.H. (1997). Bestimmung von Virulenzfaktoren Bovine Shiga-Toxin-Bildender *Escherichia coli* (STEC-) Stämme als Bewertungsgrundlage ihrer Klinischen Bedeutung für Rind und Mensch. Habilitation Ph.D. Thesis.

[24] Dow M.A., Tóth I., Alexa P., Davies M., Malik A., Oswald E., Nagy B. (2005). Predominance of afr2 and ral fimbrial genes related to those encoding the K88 and CS31A fimbrial adhesins in enteropathogenic Escherichia coli isolates from rabbits with postweaning diarrhea in Central Europe. J. Clin. Microbiol..

[25] Jinneman K.C., Wetherington J.H., Hill W.E., Omiescinski C.J., Adams A.M., Johnson J.M., Tenge B.J., Dang N.L., Wekell M.M. (1999). An oligonucleotide-ligation assay for the differentiation between Cyclospora and Eimeria spp. polymerase chain reaction amplification products. J. Food Prot..

[26] Froussard P. (1993). rPCR: A powerful tool for random amplification of whole RNA sequences. PCR Methods Appl..

[27] Faizuloev E., Mintaev R., Petrusha O., Marova A., Smirnova D., Ammour Y., Meskina E., Sergeev O., Zhavoronok S., Karaulov A. (2021). New approach of genetic characterization of group A rotaviruses by the nanopore sequencing method. J. Virol. Methods..

[28] Vilsker M., Moosa Y., Nooij S., Fonseca V., Ghysens Y., Dumon K., Pauwels R., Alcantara L.C., vanden Eynden E., Vandamme A.M. (2019). Genome Detective: An Automated System for Virus Identification from High-Throughput Sequencing Data. Bioinformatics.

[29] Beikpour F., Pellegrini F., Lanave G., Camero M., Catella C., Di Martino B., Di Profio F., Masotti C., Battistini R., Serracca L. (2023). Exploring the Astrovirome of Shellfish Matrices Using Nanopore Sequencing. Vet. Sci..

[30] Kumar S., Stecher G., Li M., Knyaz C., Tamura K. (2018). MEGA X: Molecular Evolutionary Genetics Analysis across Computing Platforms. Mol. Biol. Evol..

[31] Martella V., Ciarlet M., Camarda A., Pratelli A., Tempesta M., Greco G., Cavalli A., Elia G., Decaro N., Terio V. (2003). Molecular characterization of the VP4, VP6, VP7, and NSP4 genes of lapine rotaviruses identified in Italy: Emergence of a novel VP4 genotype. Virology.

[32] Ciarlet M., Estes M.K., Barone C., Ramig R.F., Conner M.E. (1998). Analysis of host range restriction determinants in the rabbit model: Comparison of homologous and heterologous rotavirus infections. J. Virol..

[33] Ciarlet M., Gilger M.A., Barone C., McArthur M., Estes M.K., Conner M.E. (1998). Rotavirus disease, but not infection and development of intestinal histopathological lesions, is age restricted in rabbits. Virology.

[34] Conner M.E., Estes M.K., Graham D.Y. (1988). Rabbit model of rotavirus infection. J. Virol..

[35] Petric M., Middleton P.J., Grant C., Tam J.S., Hewitt C.M. (1978). Lapine rotavirus: Preliminary studies on epizoology and transmission. Can. J. Comp. Med..

[36] Sato K., Inaba Y., Miura Y., Tokuhisa S., Matumoto M. (1982). Isolation of lapine rotavirus in cell cultures. Brief report. Arch. Virol..

[37] Thouless M.E., DiGiacomo R.F., Deeb B.J., Howard H. (1988). Pathogenicity of rotavirus in rabbits. J. Clin. Microbiol..

[38] Hall G.A., Bridger J.C., Parsons K.R., Cook R. (1993). Variation in rotavirus virulence: A comparison of pathogenesis in calves between two rotaviruses of different virulence. Vet. Pathol..

[39] DiGiacomo R.F., Thouless M.E. (1986). Epidemiology of naturally occurring rotavirus infection in rabbits. Lab. Anim. Sci..

[40] Reynoso Utrera E., Bautista Gómez L.G., Martínez Castañeda J.S., Romero Núñez C., García Rubio V.G., Aguado Almazán G.L., Hernández García P.A., Espinosa Ayala E. (2019). Análisis de la presencia de Rotavirus en conejos del Estado de México. Rev. Mex. Cienc. Pecu..

[41] Duarte A., Abade Dos Santos F.A., Fagulha T., Caetano I., Carvalho P., Carvalho J., Santos A.E., de Ayala R.P., Duarte M.D. (2025). Mixed viral infections (Rotavirus, Herpesvirus and others) in European wild rabbits. Vet. Anim. Sci..

[42] Camguilhem R., Milon A. (1989). Biotypes and O serogroups of Escherichia coli involved in intestinal infections of weaned rabbits: Clues to diagnosis of pathogenic strains. J. Clin. Microbiol..

[43] Hughes K. (2024). Endoparasites of rabbits and hares. J. Vet. Diagn. Invest..

[44] Ciarlet M., Estes M.K., Conner M.E. (1997). Comparative amino acid sequence analysis of the outer capsid protein VP4 from four lapine rotavirus strains reveals identity with genotype P[14] human rotaviruses. Arch. Virol..

[45] Martella V., Bányai K., Matthijnssens J., Buonavoglia C., Ciarlet M. (2010). Zoonotic aspects of rotaviruses. Vet. Microbiol..

[46] Zhirakovskaia E.V., Aksanova R.K., Gorbunova M.G., Tikunov A.I., Kuril’shchikov A.M., Sokolov S.N., Netesov S.V., Tikunova N.V. (2012). Genetic diversity of group A rotavirus isolates found in Western Siberia in 2007–2011. Mol. Gen. Mikrobiol. Virusol..

[47] De Grazia S., Giammanco G.M., Potgieter C.A., Matthijnssens J., Banyai K., Platia M.A., Colomba C., Martella V. (2010). Unusual assortment of segments in 2 rare human rotavirus genomes. Emerg. Infect. Dis..

[48] Grant L., Esona M., Gentsch J., Watt J., Reid R., Weatherholtz R., Santosham M., Parashar U., O’Brien K. (2011). Detection of G3P[3] and G3P[9] rotavirus strains in American Indian children with evidence of gene reassortment between human and animal rotaviruses. J. Med. Virol..

[49] Nguyen T.H., Than V.T., Thanh H.D., Kim W. (2016). Evidence of multiple reassortment events of feline-to-human rotaviruses based on a rare human G3P[9] rotavirus isolated from a patient with acute gastroenteritis. Comp. Immunol. Microbiol. Infect. Dis..

[50] Abravanel F., Lhomme S., El Costa H., Schvartz B., Peron J.M., Kamar N., Izopet J. (2017). Rabbit Hepatitis E Virus Infections in Humans, France. Emerg. Infect. Dis..

