# Supplementary material for: Complete Genome Sequencing of a G3P[14] Rabbit Rotavirus
Source: Animals (Basel). 2025 May 25;15(11):1548. doi: 10.3390/ani15111548 (PMC12153883; doi:10.3390/ani15111548)
Supplement: Supplementary file 1 [file animals-15-01548-s001.zip › animals-3510644-supplementary.pdf]

Table S1. Summary of the gross pathological findings and diagnostic screening for *Rotavirus A (*RVA), *Escherichia coli* (*E. coli*) and *Eimeria* spp. for the two cohorts of post-weaning rabbits (Group A and Group B). The severity of infection induced by *Eimeria* spp. is denoted by the number of plus signs (+).

Group	Nr.	Gross Findings	RVA (Ct value)	*E. coli*			*Eimeria spp.*
				biotype	*eae*	*afr2*	
A	1	Catarrhal enteritis, stasis of cecal tract	38.30	31	+	+	+
A	2	Catarrhal enteritis	-	31	+	+	+
A	3	Catarrhal enteritis	38.56	31	+	+	+
A	4	Catarrhal enteritis, stasis of cecal tract	-	31	+	+	+
A	5	Catarrhal enteritis, stasis of stomach	35.76	31	+	+	+
A	6	Catarrhal enteritis, stasis of cecal tract	-	31	+	+	+
A	7	Catarrhal enteritis	-	31	+	+	+
A	8	Catarrhal enteritis	36.80	31	+	+	++
A	9	Catarrhal enteritis	-	31	+	+	+
A	10	Catarrhal enteritis, stasis of cecal tract	36.95	31	+	+	++
A	11	Catarrhal enteritis, stasis of cecal tract	37.78	31	+	+	+
B	1	Catarrhal enteritis	-	31	+	+	++
B	2	Catarrhal enteritis	-	31	+	+	++
B	3	Catarrhal enteritis	29.37	31	+	+	++
B	4	Catarrhal enteritis, stasis of cecal tract	31.34	31	+	+	+++
B	5	Catarrhal enteritis	25.71	31	+	+	+++
B	6	Catarrhal enteritis	27.35	31	+	+	++
B	7	Catarrhal enteritis, stasis of stomach	29.80	31	+	+	+++
B	8	Catarrhal enteritis	25.01	31	+	+	+++
B	9	Catarrhal enteritis, stasis of stomach	15.33	31	+	+	++
B	10	Catarrhal enteritis, stasis of stomach	25.36	31	+	+	++
B	11	Catarrhal enteritis, stasis of cecal tract	24.62	31	+	+	++

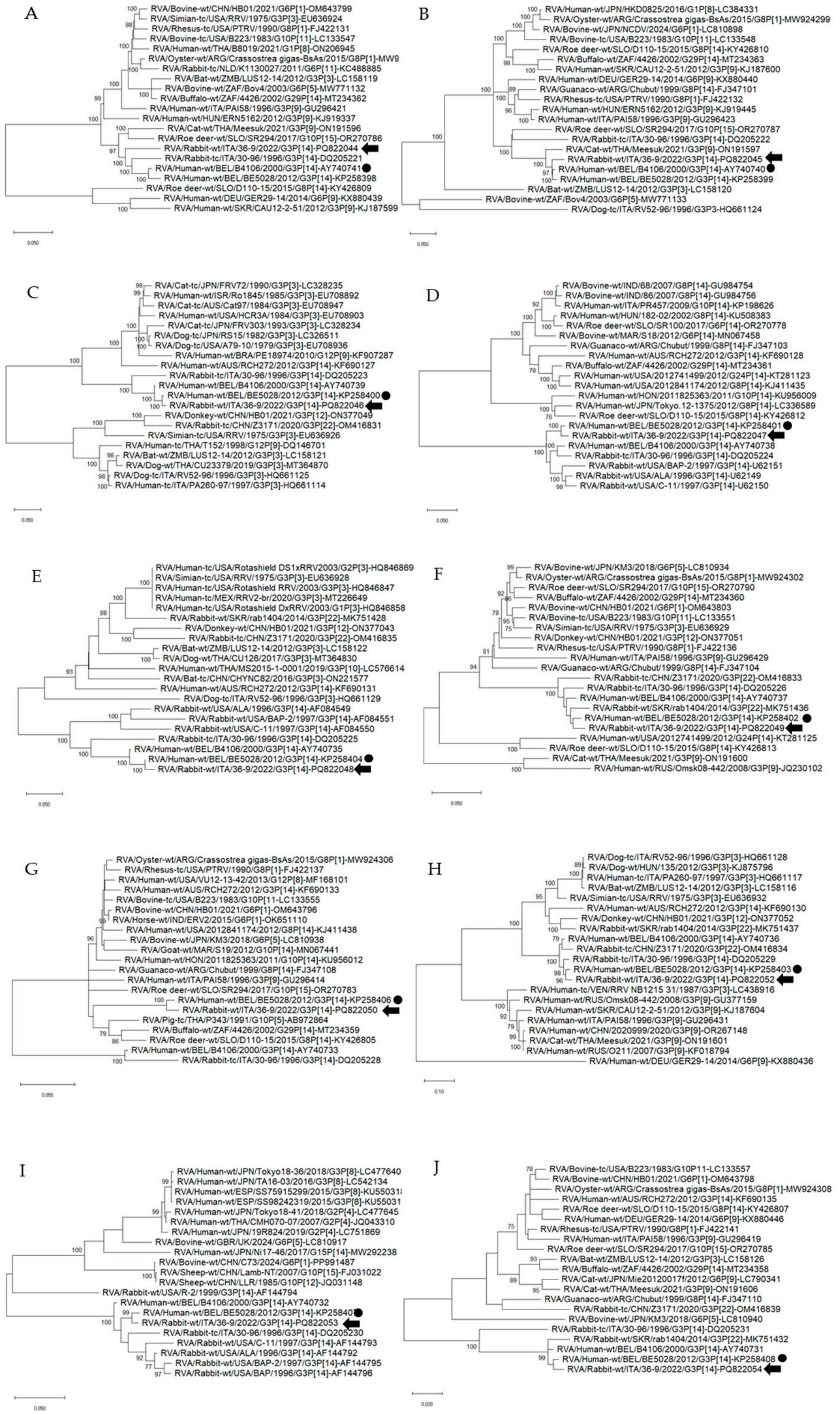

Figure S1: Phylogenetic trees of RNA-dependent RNA polymerase (VP1); capsid protein (VP2); capping enzyme (VP3); spike protein (VP4); nonstructural RNA-binding protein (NSP1); major inner capsid protein (VP6); translational regulator (NSP3); multifunctional RNA chaperone (NSP2); outer capsid protein (VP7); viral enterotoxin (NSP4); viral phosphoprotein (NSP5) nucleotide sequences of the rotavirus A strain Rabbit-wt/ITA/36-9/2022/G3P[14] compared with cognate sequences retrieved from GenBank database. Phylogeny of VP1 gene (A). Phylogeny of VP2 gene (B). Phylogeny of VP3 gene (C). Phylogeny of VP4 gene (D). Phylogeny of NSP1 gene (E). Phylogeny of VP6 gene (F). Phylogeny of NSP3 gene (G). Phylogeny of VP7 gene (H). Phylogeny of NSP4 gene (I). Phylogeny of NSP5 gene (J). The black arrow indicates the lapine RVA strain detected in this study. Black circles indicate the reference strain with the highest nt identity to Rabbit-wt/ITA/36-9/2022/G3P[14] according to Table 4. The scale bar represents the number of nt substitutions per site.
